# Therapeutic Penetrating Keratoplasty for Aspergillus Fungal Keratitis: A Case Report

**DOI:** 10.7759/cureus.103541

**Published:** 2026-02-13

**Authors:** Ioanna Gardeli, Vasileios Panagoulis

**Affiliations:** 1 Ophthalmology - Cornea and Transplant, General Hospital of Athens "Georgios Gennimatas", Athens, GRC; 2 Ophthalmology, General Hospital of Athens "Georgios Gennimatas", Athens, GRC

**Keywords:** agriculture workers, aspergillus lentulus, cyclosporine a, mycotic keratitis, ocular trauma, therapeutic penetrating keratoplasty

## Abstract

The purpose of this study was to present a case of fungal keratitis in a 35-year-old farmer successfully managed with therapeutic penetrating keratoplasty (TPK).

A 35-year-old male was referred to the emergency department with a 10-day history of a right-eye corneal ulcer. He reported a preceding ocular injury caused by a small stone during farm work. Prior to referral, he had been hospitalized at a tertiary center and treated with topical moxifloxacin and fluconazole without clinical improvement. Clinical examination revealed a corneal ulcer with an endothelial plaque, hypopyon, and fibrin in the anterior chamber. After corneal scraping, empirical treatment for fungal keratitis was initiated. Posterior segment evaluation excluded endophthalmitis. Cultures demonstrated *Aspergillus lentulus* sensitive to voriconazole. Despite intensive topical and systemic antifungal treatment (voriconazole and amphotericin B), rapid clinical deterioration occurred.* *Emergency TPK was therefore performed.

Emergency TPK was carried out. Diseased corneal tissue, hypopyon, and fibrin were removed, and copious irrigation of the anterior chamber with diluted voriconazole was performed. Instead of topical corticosteroids, postoperative topical cyclosporine A 0.5% was prescribed. Four months after surgery, the patient remains infection-free, with best-corrected visual acuity of 7^+^/10 showing continuous improvement.

TPK with meticulous anterior chamber lavage provided a definitive cure for fungal keratitis refractory to topical and systemic therapy. Postoperative administration of cyclosporine A instead of corticosteroids appears to be a safe and effective alternative for graft preservation after TPK for fungal keratitis.

## Introduction

Fungal keratitis is a vision-threatening corneal infection, even threatening the ocular integrity. It affects developing regions and individuals exposed to plants and soil material, such as agricultural workers [1,2]. Fungal keratitis represents a significant global cause of infectious corneal morbidity, with an estimated incidence of approximately one million cases annually worldwide, particularly in tropical and resource-limited settings. Despite appropriate antifungal therapy, a substantial proportion of patients (approximately 20-50%) require surgical intervention, most commonly therapeutic keratoplasty, due to inadequate medical response or progressive structural compromise of the cornea. In several series, 8-11% of the eyes may require removal [3]. *Aspergillus* species are the most frequent filamentous fungi implicated and are characterized by delayed presentation, resistance to empirical therapy, and increased risk of corneal perforation and transplantation [4,5]. *A. lentulus* is often responsible for severe mycotic keratitis [6]. Early microbiological identification and appropriate escalation of antifungal therapy remain essential to preserve globe integrity. In advanced or delayed cases, therapeutic penetrating keratoplasty (TPK) may offer a definite solution.

## Case presentation

A 35-year-old phakic male patient, a farmer by professionwas referred to our emergency department with a 10-day history of a painful right-eye corneal ulcer. The patient reported minor ocular trauma caused by a small stone during agricultural work. He had been previously hospitalized at a tertiary center, where he was treated for presumed fungal keratitis with topical moxifloxacin and fluconazole. Corneal cultures had been collected, but the results remained pending at the time of referral. No clinical improvement was observed during his initial hospitalization.

Clinical findings

**Figure 1:**
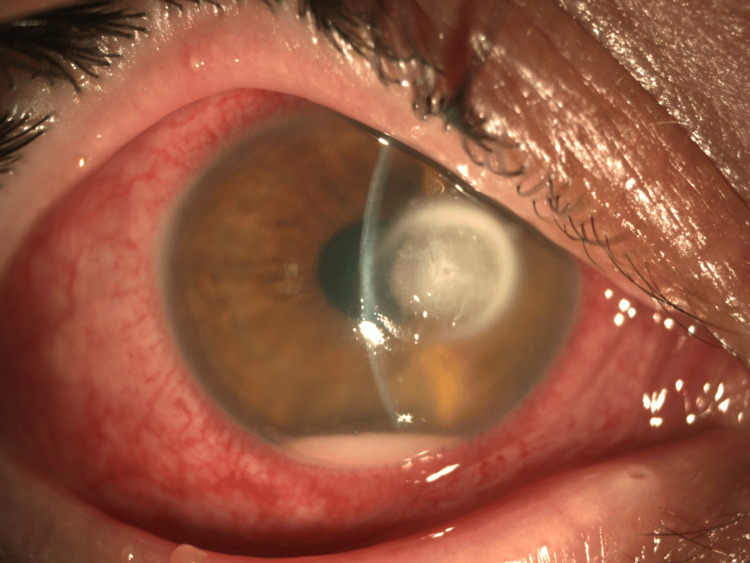
Corneal ulcer 4.0 × 4.0 mm with endothelial plaque, hypopyon, and fibrin in the anterior chamber. The lesion had a circular appearance, with a dry texture and relatively indistinct or feathery borders.

On presentation, best-corrected visual acuity in the affected eye was 20/200. Slit-lamp examination revealed conjunctival hyperemia, a 4.0 × 4.0 mm corneal ulcer, an endothelial plaque, 1-mm hypopyon, and anterior chamber fibrin reaction. The lesion had a circular appearance, with a dry texture and relatively indistinct or feathery borders. The deep stromal involvement was also suggestive of fungal keratitis (Figure 1).

Investigations

Repeat corneal scrapings for culture and polymerase chain reaction (PCR) for fungal pathogens were obtained. B-scan ultrasonography and fundoscopy ruled out endophthalmitis, while orbital CT excluded the presence of an intraocular foreign body or orbital spread of infection. Broad-spectrum antimicrobial and antimycotic therapies were initiated, including topical amikacin, vancomycin, and voriconazole, along with oral doxycycline and intravenous ceftazidime and voriconazole. Both initial and repeat cultures, as well as PCR, identified *A. lentulus* sensitive to voriconazole and amphotericin B.

Management

**Figure 2:**
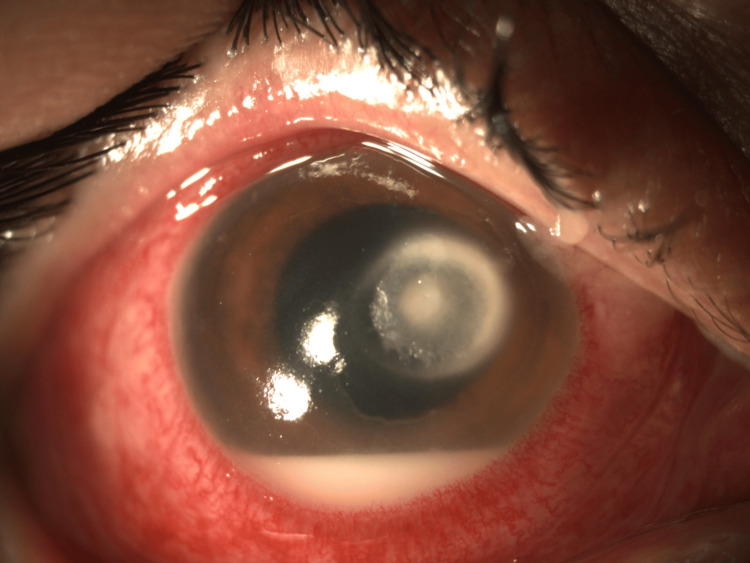
Clinical deterioration prior to surgery: corneal ulcer 7.5 × 7.5 mm with hypopyon progression and fibrin extending from the iris root to the pupillary edge.

Despite escalation to intravenous voriconazole and topical voriconazole and amphotericin B, rapid clinical deterioration occurred, with ulcer enlargement to 7.5 × 7.5 mm, hypopyon depth increasing to 2.5 mm, and fibrin extending to the pupillary margin (Figure 2). Given the absence of response to conservative therapy, emergency TPK was performed under general anesthesia.

**Figure 3:**
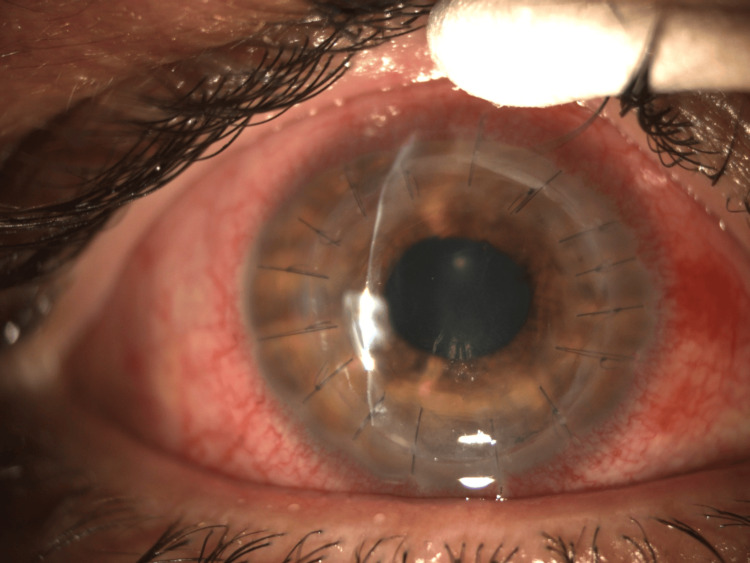
1st day postoperatively, the hypopyon and fibrin were removed. The corneal graft was secured with 16 interrupted 10-0 nylon sutures.

Diseased corneal tissue was excised, and an open-sky approach allowed meticulous removal of fibrin and hypopyon. Anterior chamber irrigation with diluted voriconazole (3 mg/mL) was supplemented by mechanical disruption of adherent inflammatory material. The corneal graft was secured with 16 interrupted 10-0 nylon sutures (Figure 3).

Postoperatively, the patient received topical ofloxacin, voriconazole, and cyclosporine A 0.5% instead of topical steroids, along with oral voriconazole.

Outcome and follow-up

**Figure 4:**
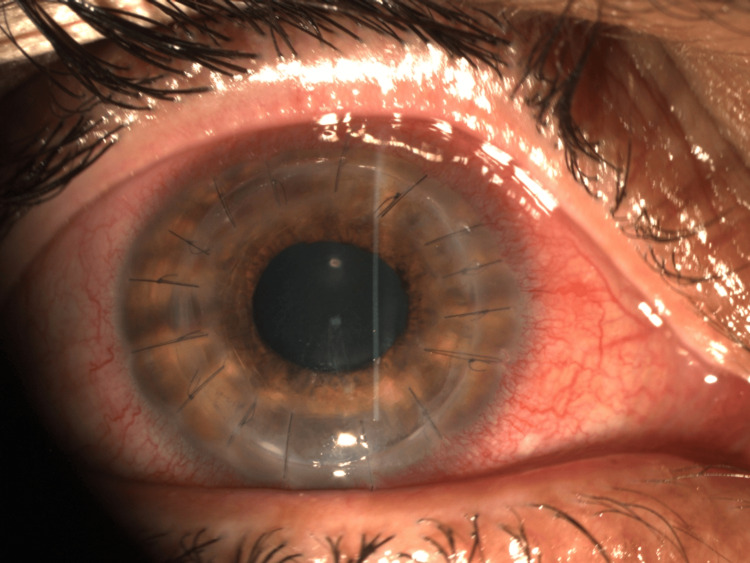
10 days postoperatively, the graft was clear and remained free of infection. The sutures were all in situ.

**Figure 5:**
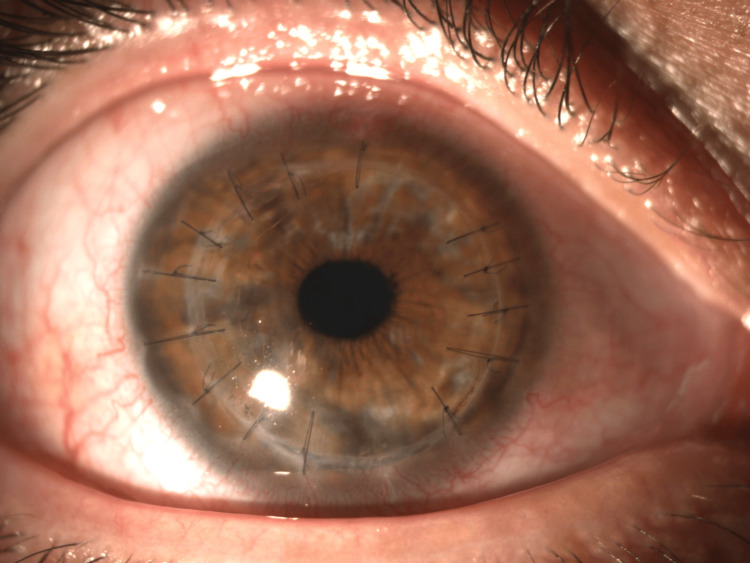
Postoperative image at four and a half months demonstrating a clear, infection-free corneal graft, with two sutures removed due to loosening.

At 10 days (Figure 4) and at 4.5 months after surgery (Figure 5), the corneal graft remained clear, the eye was infection-free, and best-corrected visual acuity improved to 20/30 with continued improvement at subsequent reviews.

## Discussion

Fungal keratitis remains a major cause of corneal blindness globally, even leading to ocular enucleation, especially for agricultural workers exposed to plant material trauma [1,2,4]. *A. lentulus* is a cryptic pathogen with high resistance to drugs, which is explained by azole and polyene resistance mechanisms [6].

Because of a prolonged inaccessibility of natamycin in our setting, voriconazole is the preferred available treatment agent due to its superior corneal and aqueous penetration, although in cases with deep stromal involvement and delayed presentation, persistent treatment resistance is observed [5,7-9]. Amphotericin B was used in addition to voriconazole in an attempt to control the infection, but it was ineffective.

Clinical deterioration with progressive ulceration is an indication for emergency TPK. Infection debridement, intraoperative antifungal irrigation, sustained postoperative antifungal therapy, and cyclosporine use instead of corticosteroids are key factors for surgical success [10,11].

Topical corticosteroids remain controversial in the early postoperative period following therapeutic keratoplasty for fungal keratitis, due to their potential to exacerbate residual fungal infection or promote recurrence. For this reason, topical cyclosporine A has been used as an alternative immunomodulator, providing anti-inflammatory effects and graft rejection control without the same level of fungal proliferation risk. Experimental data suggest that cyclosporine A exerts T-cell suppression without significant impact on fungal growth kinetics, making it a safer bridge therapy until the eye is microbiologically quiet and steroid therapy can be safely introduced [12-14]. Chatterjee and Agrawal demonstrated that postoperative cyclosporine A use reduced early graft rejection without increasing fungal recurrence, supporting its value in high-risk infectious keratoplasty settings. Clinical series also report improved graft clarity and tolerance when cyclosporine A 0.1% is used selectively in complex fungal keratitis cases where clinicians avoid corticosteroids in the early healing phase [15,16].

Recent analyses confirm that timely TPK improves infection control, maintains globe integrity, and provides meaningful visual recovery [11,15]. Immediate surgical intervention is required in the presence of corneal perforation, impending perforation, or rapid stromal melt. Early TPK should be considered when there is no meaningful clinical improvement after approximately 7-14 days of intensive, organism-directed antifungal therapy, particularly in cases with progressive deep stromal involvement, persistent or increasing hypopyon, or large central ulcers. Delaying surgery until overt structural collapse is associated with poorer anatomical outcomes and higher complication rates [17-19].

## Conclusions

This case highlights the aggressive clinical course of *A. lentulus* keratitis following agricultural trauma. Despite culture-guided antifungal therapy, rapid deterioration necessitated emergency penetrating keratoplasty. Prompt microbiologic diagnosis, intraoperative antifungal intervention, and postoperative immunomodulation resulted in infection eradication, graft clarity, and functional visual recovery. Early surgical escalation remains essential in drug-resistant fungal keratitis. Cyclosporine A represents a practical interim immunomodulatory strategy in fungal keratitis management following TPK, balancing infection safety with control of alloimmune inflammation.
